# Antibacterial and Antibiofilm Efficacy of Thyme (*Thymus vulgaris* L.) Essential Oil against Foodborne Illness Pathogens, *Salmonella enterica* subsp. *enterica* Serovar Typhimurium and *Bacillus cereus*

**DOI:** 10.3390/antibiotics12030485

**Published:** 2023-02-28

**Authors:** Daniela Sateriale, Giuseppina Forgione, Giuseppa Anna De Cristofaro, Chiara Pagliuca, Roberta Colicchio, Paola Salvatore, Marina Paolucci, Caterina Pagliarulo

**Affiliations:** 1 Department of Science and Technology, University of Sannio, Via F. De Sanctis snc, 82100 Benevento, Italy; 2 Department of Molecular Medicine and Medical Biotechnologies, University of Naples Federico II, Via S. Pansini 5, 80131 Naples, Italy; 3 CEINGE-Biotecnologie Avanzate s.c.ar.l., Via G. Salvatore 486, 80145 Naples, Italy

**Keywords:** thyme essential oil, antibacterial agent, antibiofilm activity, natural food preservatives, foodborne illness pathogens

## Abstract

Nowadays, the wide spread of foodborne illness and the growing concerns about the use of synthetic food additives have shifted the focus of researchers towards essential oils (EOs) as possible antimicrobials and preservatives of natural origin. Thanks to their antimicrobial properties against pathogenic and food spoilage microorganisms, EOs have shown good potential for use as alternative food additives, also to counteract biofilm-forming bacterial strains, the spread of which is considered to be among the main causes of the increase in foodborne illness outbreaks. In this context, the aim of this study has been to define the antibacterial and antibiofilm profile of thyme (*Thymus vulgaris* L.) essential oil (TEO) against widespread foodborne pathogens, *Salmonella enterica* subsp. *enterica* serovar Typhimurium and *Bacillus cereus*. TEO chemical composition was analyzed through gas chromatography-mass spectrometry (GC-MS). Preliminary in vitro antibacterial tests allowed to qualitatively verify TEO efficacy against the tested foodborne pathogens. The subsequent determination of minimal inhibitory concentration (MIC) and minimal bactericidal concentration (MBC) values allowed to quantitatively define the bacteriostatic and bactericidal effects of TEO. To evaluate the ability of essential oils to inhibit biofilm formation, a microplate assay was performed for the bacterial biofilm biomass measurement. Results suggest that TEO, rich in bioactive compounds, is able to inhibit the growth of tested foodborne bacteria. In addition, the highlighted in vitro anti-biofilm properties of TEO suggest the use of this natural agent as a promising food preservative to counteract biofilm-related infections in the food industry.

## 1. Introduction

Foodborne illness encompasses a wide spectrum of illnesses defined by the WHO’s Department of Food Safety, Zoonoses and Foodborne Diseases (FOS) as a growing public health problem worldwide [1]. In recent years, most of the foodborne outbreaks in Europe and the United States were related to the consumption of contaminated meat and meat products; fresh processed products of pig meat were the most frequently involved category, along with chicken meat [2]. Meat and meat products contain essential amino acids, B-group vitamins, minerals and other nutrients ideal both for human nutrition and microbial growth [3]. Fresh and transformed meat are rich substrates that strongly support significant microbial growth, since they have nutrients, pH values and water contents generally compatible with the growth of a large number and variety of microorganisms [4]. Therefore, meat and meat products are easily perishable foods and are highly susceptible to the growth of microorganisms, including spoilage microorganisms and foodborne pathogens [5] causing outbreaks which severely affect public health and the economy [6].

The pathogens of great concern for foodborne illness outbreaks mainly include *Salmonella* spp., enterohemorrhagic *Escherichia coli*, *Listeria monocytogenes*, *Bacillus* spp., *Staphylococcus aureus* and *Clostridium* spp. [4,7].

*Salmonella* spp. is among the most common etiological agents of human gastroenteritis, frequently isolated from both the mammal’s gut and environment. Effluents, floors and walls of contaminated abattoir are considered major sources of *Salmonella* species [8,9]. Several serotypes of *Salmonella enterica* such as *Salmonella enterica* subsp. *enterica* serovar Typhimurium are particularly dangerous because they can cause food poisoning and develop biofilms that are difficult to remove [10].

Among other bacterial pathogens causing foodborne illnesses, *Bacillus cereus* is well-known. It is spread both in the environment and in raw foods, such as meat, rice and vegetables. The inadequate cooling after heat treatment is indicated as the main factor contributing to the spread of *B. cereus* in the above-mentioned foods [11]. This microorganism is of particular concern for the food industry, causing food safety issues due to the formation of spores, emetic toxins and biofilms causing diarrhea [12].

The ability of foodborne strains to develop biofilms on different surfaces enhances their persistence in different environments by increasing their physical and chemical resistance [13]. Biofilm-forming bacteria are able to adhere to a wide range of surfaces, including stainless steel, plastic, glass and also food. The adhesion process occurs in a few minutes, until the development of mature biofilms in some hours or days [14], causing major challenges for the food industry [15].

The use of safe and effective preservation methods that allow to counteract the spread of foodborne illness outbreaks and prevent the deterioration of meat products is crucial. Heating, chilling and packaging are some of the current methods for meat and meat product preservation, together with high pressure and ionizing radiation [4]. In addition, the use of chemical preservatives and additives, such as nitrites and nitrates, allow to prevent the spoilage caused by foodborne microorganisms and pathogenic bacteria in meat products [16]. However, the increasing use of synthetic additives in food has raised many carcinogenic and toxic problems and has been related to allergy episodes [4,17,18,19]. For these reasons, consumers today demand food free of artificial substances, such as antimicrobial and chemically synthesized food preservatives, as they are perceived as harmful to health. Current trends for products without synthetic preservatives have led to the search for natural antimicrobial compounds. There has been thorough research on natural food additives with a broad spectrum of antimicrobial activities, capable of improving the microbiological quality and the shelf-life of perishable foods. Aromatic plants and their derivatives, such as essential oils (EOs), have attracted considerable interest [20,21,22,23,24]. Numerous studies demonstrated that essential oils employed as preservatives possess in vitro antimicrobial properties [25,26]. For instance, results of recent research demonstrated the antimicrobial activity of thyme essential oil against several foodborne pathogens [27,28,29], thus encouraging their potential use to reduce pathogenic bacteria spread and for the extension of food products’ shelf-life [30]. Several studies are underway to evaluate the effectiveness of essential oils when added to foods [31,32], while studies on the application of EOs to prevent biofilm production on food and environmental surfaces have recently increased [33,34,35].

In this frame, the present study aims to evaluate the in vitro antibacterial effects of thyme essential oil (TEO), form *Thymus vulgaris* L., against important and common causative agents of food infections. Even if TEO is well-known for its ability to inhibit the growth of both Gram negative and Gram positive bacteria [36,37,38], a significant variability may exist among the in vitro antibacterial effects of the same type of EO, depending both on EO chemical composition and variable susceptibility by tested microbial isolates. In addition, foodborne isolates of the same species could present a different susceptibility/resistance to natural extracts [39,40,41]. In this regard, this work aims to confirm the presence of antimicrobial molecules in the tested Italian TEO and to increase the number of scientific evidences demonstrating the sensitivity of important foodborne illness etiological agents to TEO. In particular, the in vitro antibacterial activity of TEO was evaluated against two meat isolates, i.e., *S.* Typhimurium and *Bacillus cereus*. Considering that recent studies showing TEO antibiofilm effects are still few, TEO was tested against foodborne pathogenic isolates both in planktonic and biofilm form, with the future prospect to use it in food preservation to control the safety and quality of meat products along the entire food chain and biofilm formation associated with foodborne pathogens.

## 2. Results and Discussion

### 2.1. Chemical Composition of Thyme Essential Oil

The quality and authenticity of thyme essential oil (TEO) tested in this study were evaluated by the manufacturer according to the European Pharmacopoeia (Ph. Eur.) recommendations, by checking the purity of plant raw materials (*Thymus vulgaris* L.) and by measuring quality markers after extraction. In particular, panel sensory analysis allowed the examination of TEO organoleptic properties (odor, color, aspect), while physical analysis (i.e., density and moisture content measuring) allowed to evaluate its purity (>95%), along with the absence of water, and to define TEO as free from foreign bodies or extraneous matter, and from impurities of the raw material itself. These simple, cheap and fast analyses allowed the identification of any adulteration, but chemical characterization analysis by gas chromatography mass spectrometry was necessary to confirm TEO specific fingerprint.

**Figure 1:**
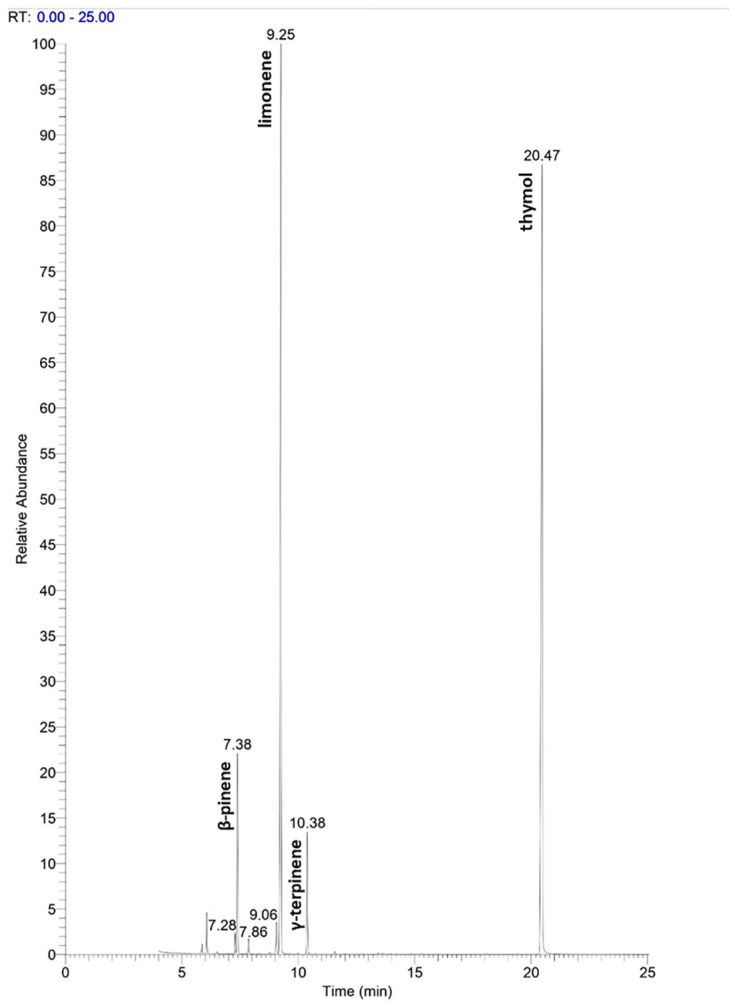
Gas chromatogram of the tested thyme essential oil. The most abundant components were identified through GC-MS analysis by Thermo Scientific TRACE 1310 GC system coupled to an ITQ 900 mass spectrometer and are assigned in the figure. RT, retention time.

TEO chemical composition is shown in Table 1. Seven main volatile compounds, representing 98.838 ± 0.99% of the total detected constituents, were identified by gas chromatography mass spectrometry analyses in TEO (Figure 1).

Thymol (44.435 ± 0.22%), limonene (39.391 ± 0.20%), β-pinene (7.177 ± 0.04%) and γ-terpinene (4.405 ± 0.02%) were identified as the major constituents. The other components, i.e., sabinene, α-phellandrene and o-cymene, were present in a total amount of less than 4% (3.430 ± 0.51%).

Essential oils (EOs) are complex matrices with extremely variable composition and properties. It is known that the chemical constituents of EOs may vary depending on several factors, including harvest season, habitat, drying processes of plant raw material, extraction techniques, storage time and others [42,43]. Due to this variability, the determination of the TEO constituent profile was necessary to confirm the presence of bioactive molecules, including antibacterial compounds. In particular, the chemical composition of TEO in our study is found to be comparable to previous literature studies reporting thymol, limonene, β-pinene and γ-terpinene as major compounds in TEO [44,45,46,47,48,49]. Similar studies reported that thymol percentage ranging from 12% to 71% for *Thymus vulgaris* EO really contributes to its antimicrobial activity [46,47].

### 2.2. In Vitro Antibacterial Activity of Thyme Essential Oil against Salmonella enterica subsp. Enterica Serovar Typhimurium and Bacillus cereus Food Isolates

**Figure 2:**
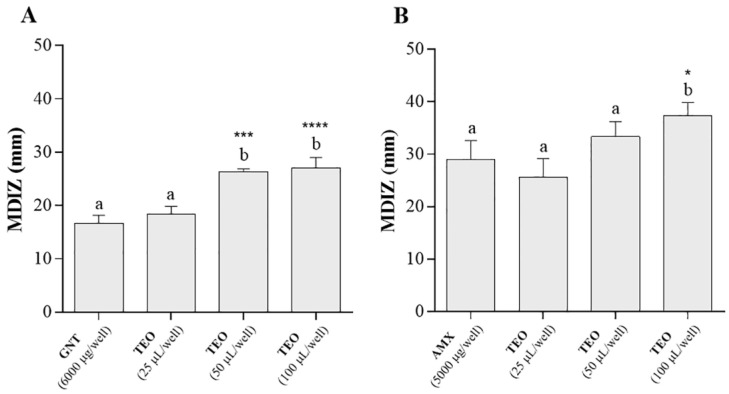
In vitro antibacterial activity of thyme essential oil against *S.* Typhimurium ST1 (**A**) and *B. cereus* BC3 (**B**) food isolates. Results were obtained by agar well diffusion method; triplicate assays with independent cultures. The mean diameters of inhibition zone, reported as mean values ± standard deviation (expressed in mm), are graphically represented. One-way ANOVA test was performed to evaluate statistical significance. Bars comparison with positive control bar (absence of TEO) was analyzed by Dunnett’s post hoc test (*p* < 0.05), using asterisks to indicate statistical significance respect to the positive control (**** *p* < 0.0001; *** *p* < 0.001; * *p* < 0.05). Tukey’s post hoc test (*p* < 0.05) allowed to examine the statistical significance for multiple comparisons between bars. Different letters (a, b) indicate significant differences between bars; bars with no significant differences receive the same letter. MDIZ, mean diameter of the inhibition zone; TEO, thyme essential oil; GNT, gentamicin; AMX, amoxicillin.

TEO exhibited an appreciable inhibitory activity against both *S.* Typhimurium and *B. cereus* bacterial meat isolates, as demonstrated by the inhibition zone of bacterial growth estimated through the agar well diffusion method. Figure 2 shows the mean diameter of the inhibition zone (MDIZ) of bacterial growth exerted by TEO against *S.* Typhimurium ST1 (Figure 2A) and *B. cereus* BC3 (Figure 2B) isolates.

The MDIZ values ranged between 18.33 ± 1.25 mm (vs. *S.* Typhimurium ST1 at the volume of 25 µL/well) and 37.33 ± 2.05 mm (vs. *B. cereus* BC3 at the volume of 100 µL/well). Gentamicin and amoxicillin, tested as positive controls, showed antibacterial efficacy against the isolates, with MDIZ values of 16.67 ± 2.25 mm and 29.00 ± 2.94 mm, respectively; no effects were observed for the negative control. The MDIZ values measured around the wells filled with TEO (at the volumes of 50 µL/well and 100 µL/well against *S.* Typhimurium ST1 and at the volumes of 100 µL/well against *B. cereus* BC3) were significantly higher with respect to MDIZ values measured around the wells filled with the antibiotics gentamicin and amoxicillin. The in vitro antibacterial activity of TEO was also confirmed by quantitative assays. Table 2 shows values of minimum inhibitory concentration (MIC) and minimum bactericidal concentration (MBC).

Tested TEO was able to exert both bacteriostatic and bactericidal effects against *S.* Typhimurium ST1 and *B. cereus* BC3 isolates, with a lower sensitivity of the Gram-negative isolate of *S.* Typhimurium to TEO, compared to the Gram-positive isolate of *B. cereus*. These results are partly in line with recent data in the literature. The significant in vitro antimicrobial activity of TEO has been demonstrated by different studies against various food pathogens, including Gram-positive bacteria, such as *Bacillus cereus* [47], and also against several serotypes of *S.* Typhimurium spp. [50]. In particular, the recent study conducted by Gonzalez et al. (2021) confirmed the in vitro bactericidal activity of TEO against *S.* Typhimurium, an important Gram negative pathogen and causative agent of food outbreaks [51]. In addition, obtained results are particularly significant given that the Italian tested TEO resulted to be more effective compared to other previous studies which tested this essential oil against the same bacterial species. For example, in the study by Valizadeh et al. (2016), the values of the inhibition zone diameter (mm) obtained by testing *Thymus vulgaris* essential oil using the agar well diffusion method were shown to be significantly lower both against *S.* Typhimurium (15 mm) and *B. cereus* (17 mm) isolates [52], compared to Italian TEO tested in this study at the same volume (100 μL) and with the same method. Moreover, MIC for TEO against *S.* Typhimurium ST1 food isolate indicates more significant antimicrobial effects of tested essential oil than those obtained by Penalver et al. (2005); the MIC value (4% *v*/*v*) for thyme essential oil against *S.* Typhimurium was two-fold higher, indicating lower in vitro antimicrobial effects [53]. In the study of Turgis et al. (2012) no activity was observed for *Thymus vulgaris* essential oil against *S.* Typhimurium at the concentration of 4000 ppm [54]. Finally, another recent study reports a five-fold higher MIC value against *B. cereus* (5% *v*/*v*) than that obtained by testing TEO against *B. cereus* BC3 isolate [55], thus demonstrating the more significant activity of tested Italian TEO against this foodborne pathogen.

TEO antimicrobial activity depends on its chemical composition. Synergistic interactions between bioactive compounds are responsible for specific antimicrobial mechanisms of action. According to Kang et al. (2018), TEO rich in p-cymene, thymol and γ-terpinene showed the ability to inhibit the growth of *B. cereus* by causing membrane damage, alterations in cell morphology and a decrease in the intracellular pool of ATP [56]. The hyper-permeabilization of the bacterial cell membrane, resulting in the loss of membrane potential and collapse of proton pumps and depletion of ATP, with consequent delays or inhibitions of microbial growth, could be the main mechanism of action of TEO against *Salmonella* spp. [35].

### 2.3. In Vitro Antibiofilm Activity of Thyme Essential Oil against Salmonella enterica subsp. Enterica Serovar Typhimurium and Bacillus cereus Food Isolates

The anti-biofilm activity of TEO was evaluated against *S.* Typhimurium and *B. cereus* food isolates by the tissue culture plate method to assess the impact of this natural agent upon biofilm formation in the food industry.

**Figure 3:**
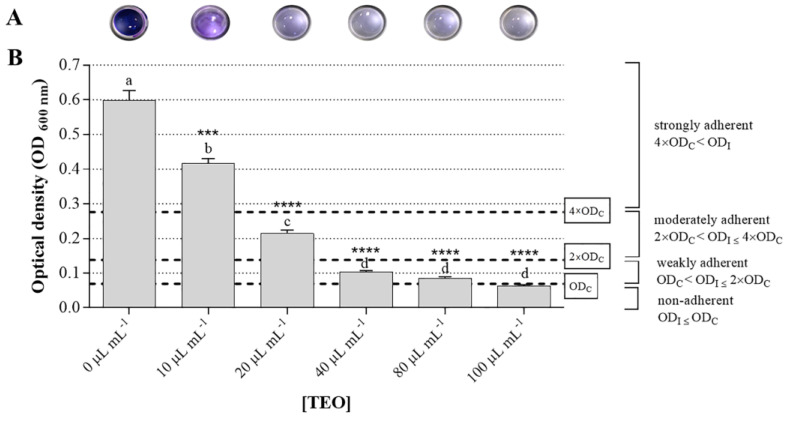
Adherence levels of *S.* Typhimurium ST1 food isolate. (**A**) Biofilm biomass at the bottom of multi-well plates in the presence and absence of increasing concentrations (10, 20, 40, 80, 100 µL mL^−1^) of TEO. (**B**) Optical density (OD) values, detected by absorbance reading at a wavelength of 600 nm with a microplate reader, of bacterial biofilms developed by *S.* Typhimurium ST1 in absence and in the presence of increasing concentrations (10, 20, 40, 80, 100 µL mL^−1^) of TEO. The comparison with negative control, represented by the broth medium, allowed to determine the level of adherence for each experimental condition. One-way ANOVA test was performed to evaluate statistical significance. Bars comparison with positive control bar (absence of TEO) was analyzed by Dunnett’s post hoc test (*p* < 0.05), using asterisks to indicate statistical significance with respect to the positive control (**** *p* < 0.0001; *** *p* < 0.001). Tukey’s post hoc test (*p* < 0.05) allowed to examine the statistical significance for multiple comparisons between bars. Different letters (a–d) indicate significant differences between bars; bars with no significant differences receive the same letter. TEO, thyme essential oil; OD_I_, isolates optical density, OD_C_, negative control optical density.

**Figure 4:**
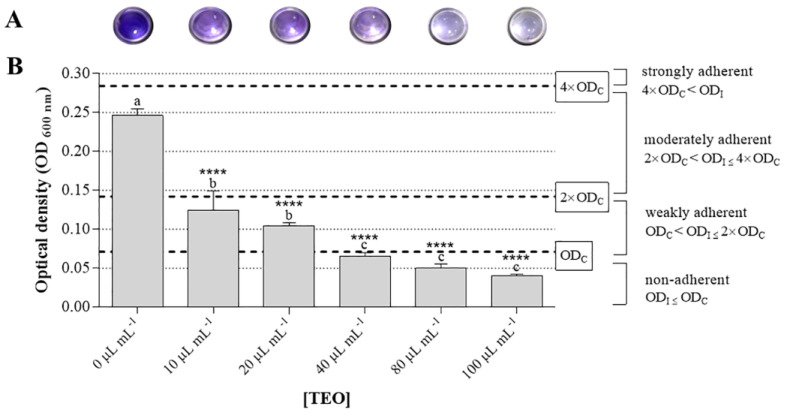
Adherence levels of *B. cereus* BC3 food isolate. (**A**) Biofilm biomass at the bottom of multi-well plates in the presence and absence of increasing concentrations (10, 20, 40, 80, 100 µL mL^−1^) of TEO. (**B**) Optical density (OD) values, detected by absorbance reading at a wavelength of 600 nm with a microplate reader, of bacterial biofilms developed by *B. cereus* BC3 in the presence and absence of increasing concentrations (10, 20, 40, 80, 100 µL mL^−1^) of TEO. The comparison with negative control, represented by the broth medium, allowed to determine the level of adherence for each experimental condition. One-way ANOVA test was performed to evaluate statistical significance. Bars comparison with positive control bar (absence of TEO) was analyzed by Dunnett’s post hoc test (*p* < 0.05), using asterisks to indicate statistical significance with respect to the positive control (**** *p* < 0.0001). Tukey’s post hoc test (*p* < 0.05) allowed to examine the statistical significance for multiple comparisons between bars. Different letters (a, b, c) indicate significant differences between bars; bars with no significant differences receive the same letter. TEO, thyme essential oil; OD_I_, isolates optical density, OD_C_, negative control optical density.

In vitro microbiological tests showed that TEO caused a significant inhibition of biofilm biomass of both *S.* Typhimurium and *B. cereus* food isolates compared to untreated controls, as reported in Figure 3A and in Figure 4A, respectively.

Optical density (OD_600nm_) values were measured for biofilms grown in the absence and in the presence of increasing concentrations of TEO (10, 20, 40, 80, 100 µL mL^−1^). Comparison with the negative control values, represented by sterile broth growth medium, allowed for the determination of the adhesion level for each test condition, enabling the classification of microbial isolates as non-adherent, weakly adherent, moderately adherent and strongly adherent.

In detail, in the absence of TEO (0 µL mL^−1^), and at the concentration of 10 µL mL^−1^, *S.* Typhimurium ST1 isolate showed strong adherence (4 × OD_C_ < OD_I_), while in the presence of increasing concentrations of oil, there was a gradual decrease in adhesion, until the absence of adherence at the concentration of 100 µL mL^−1^ of TEO (OD_I_ ≤ OD_C_) (Figure 3B), identified as the minimum biofilm inhibition concentration (MBIC) value for the tested oil against *S.* Typhimurium isolate. *B. cereus* BC3 isolate showed strong adherence (4 × OD_C_ < OD_I_) in the absence of thyme essential oil (0 µL mL^−1^), while in the presence of increasing concentrations of TEO, there was a gradual decrease in adhesion, up to the absence of adhesion at the concentration of 40 µL mL^−1^ of TEO (OD_C_ ≤ OD_I_) (Figure 4B). Therefore, the concentration of 40 µL mL^−1^ can be indicated as the minimum biofilm inhibition concentration (MBIC) for TEO against *B. cereus* meat isolate.

**Figure 5:**
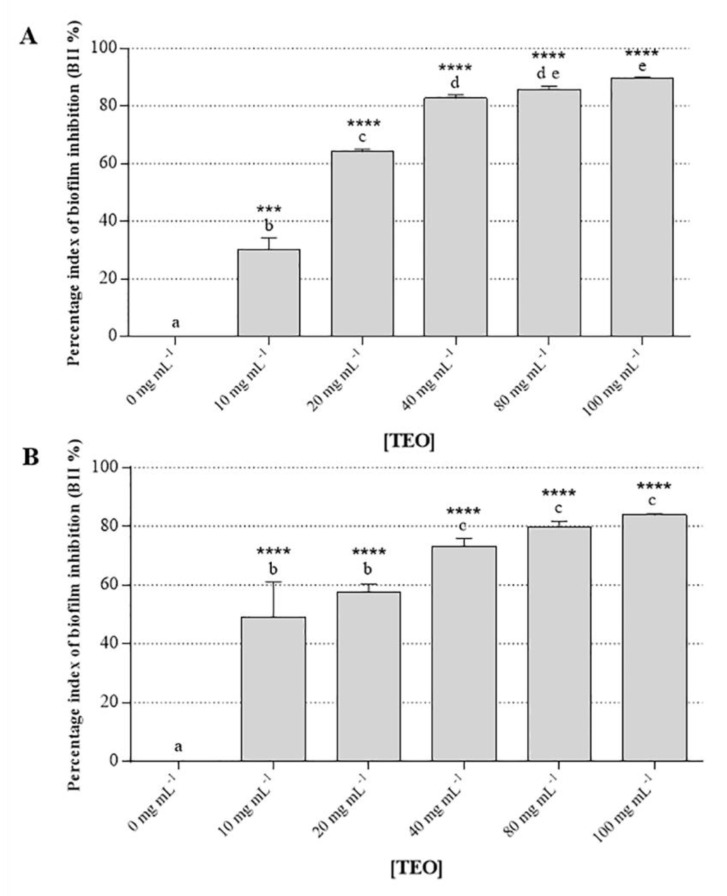
Inhibitory effect of thyme essential oil against biofilm formation by *S.* Typhimurium ST1 (**A**) and *Bacillus cereus* BC3 (**B**) food isolates. Graphs show the percentage inhibition values of biofilms in the presence and absence of increasing concentrations of TEO (10, 20, 40, 80, 100 µL mL^−1^). One-way ANOVA test was performed to evaluate statistical significance. Bars comparison with positive control bar (absence of TEO) was analyzed by Dunnett’s post hoc test (*p* < 0.05), using asterisks to indicate statistical significance with respect to the positive control (**** *p* < 0.0001; *** *p* < 0.001). Tukey’s post hoc test (*p* < 0.05) allowed to examine the statistical significance for multiple comparisons between bars. Different letters (a, b, c, d, e) indicate significant differences between bars; bars with no significant differences receive the same letter. TEO, thyme essential oil.

At increasing concentrations of TEO, a progressive increase in the percentage of biofilm inhibition was observed, for both *S.* Typhimurium (Figure 5A) and *B. cereus* (Figure 5B) food isolates.

In particular, TEO was able to inhibit biofilm formation by *S.* Typhimurium ST1 at the concentration of 10 µL mL^−1^, with an inhibition percentage of 30.03 ± 3.52%, showing a progressive increase in the percentage of biofilm inhibition with increasing TEO concentrations, reaching the inhibition percentage of 89.62 ± 0.32% at the concentration of 100 µL mL^−1^ (Figure 5A); in the absence of TEO, there was no inhibition of biofilm formation for *S.* Typhimurium food isolate. These results are really promising, compared with results of other studies which demonstrated the antibiofilm effect of thyme essential oil against *Salmonella* spp., with an inhibition percentage of biofilm formation exceeding 60% but generally less than 80% [35,57,58]. This effect could be attributed to the ability of TEO monoterpenes to spread through the exopolysaccharide matrix (EPS) of bacterial biofilms, interfering with key events in the biofilm formation process, such as adhesion protein production [35].

Regarding the tested *B. cereus* meat isolate, there was no inhibition of biofilm formation in the absence of TEO. TEO inhibited the biofilm formation at the concentration of 10 µL mL^−1^ (inhibition percentage of 49.11 ± 9.73%), even if the food isolate was still classifiable as adherent. At the concentration of 100 µL mL^−1^, the percentage of biofilm inhibition reached the value of 83.85 ± 0.28% (Figure 5B). In the absence of TEO, there was no inhibition of biofilm formation for both *S.* Typhimurium and *B. cereus* food isolates. Other previous studies demonstrated that TEO exerts a significant inhibitory effect on the formation of biofilm by *B. cereus*, although the values of inhibition percentage are lower than those reported in this study and the mechanisms have not yet been fully understood [56,59].

## 3. Materials and Methods

### 3.1. Thyme Essential Oil (TEO)

Thyme essential oil (TEO) was provided by the Italian manufacturing company Alta Profumeria S.r.l. (Durazzano, Benevento, Italy) in collaboration with the University of Sannio for several scientific research projects. It was obtained from plant aerial parts (leaves and flowers) of *Thumus vulgaris* L. by the steam distillation method and it was stored at 0–4 °C in the dark until further use.

### 3.2. Gas Chromatography-Mass Spectrometry Analysis of TEO

GC-MS analysis was carried out by Thermo Scientific TRACE 1310 GC system coupled to an ITQ 900 mass spectrometer (Thermo Fisher Scientific, Waltham, MA, USA). An HP-5MS capillary column (30 m × 0.25 mm, 0.25 μm film thickness) was used. Analyses were performed according to Alsaraf et al. (2020) [60], with slight modifications. The maximum elution temperature was 350 °C, with helium as the carrier gas (flow rate of 1 mL min^−1^). Injection temperature was set to 290 °C, while 270 °C was the set temperature for the ion source. The chemical ionization energy of 70 eV was used for MS. Mass spectral data collection in full-scan mass was carried out between 40 and 550 amu. TEO was diluted in hexane (1:10 dilution ratio) and an aliquot of sample (2 μL) was injected (150:1 split ratio). Oven temperature was programmed at 60 °C, with a constant gradually increasing rate of 3 °C min^−1^ until 280 °C. Compounds were identified using NIST MS Search Software (version 2.0). Identification was based on retention indices relatives to n-alkanes and computer matching by comparing query mass spectra with reference mass spectra in the Wiley 8 mass spectral libraries (USA National Institute of Science and Technology software 2.0) of the GC/MS data system. Analyses were performed in duplicate and the percentage of each identified compound was estimated by dividing its mean area by the total chromatogram area.

### 3.3. Meat Bacterial Isolates and Growth Conditions

TEO was tested for its antibacterial effects against *Salmonella enterica* subsp. *enterica* serovar Typhimurium and *Bacillus cereus* meat isolates in the Laboratory of Microbiology of the Department of Science and Technology, University of Sannio. Bacterial isolates used in this study were denominated *S.* Typhimurium ST1 and *B. cereus* BC3. They were isolated from samples of minced chicken meat (*S.* Typhimurium ST1) and minced pig meat (*B. cereus* BC3) and identified by differential and chromogenic selective media. In particular, the selective and chromogenic and differential media Xylose Lysine Desoxycholate Agar (CONDA, Madrid, Spain) and *Bacillus* ChromoSelect Agar (Sigma-Aldrich S.r.l., Milano, Italy), with the addition of Polymyxin B Selektiv-Supplement (Cat. No. P9602, Sigma-Aldrich S.r.l., Milano, Italy), were used for in vitro growth and identification of *S.* Typhimurium and *B. cereus* isolates, respectively. After Gram staining (Gram staining kit, Sigma-Aldrich S.r.l., Milano, Italy), the observation by microscopy Motic B1-223 (Thermo Fisher Scientific, Waltham, MA, USA) allowed to complete the phenotypic identification. *S.* Typhimurium ST1 isolate was shown to be susceptible to β-lactam antibiotics (amoxicillin and ampicillin) and chloramphenicol, as well as gentamicin, chosen as the positive control (Supplemenatry Material Table S1). The sensitivity profile of *B. cereus* BC3 isolate (Table S1) shows its susceptibility to chloramphenicol, gentamicin and β-lactam antibiotics, especially to amoxicillin, selected as the control for following antimicrobial tests. Meat bacterial isolates were grown at 37 °C for 24 h in aerobic conditions, on the non-selective medium Luria Bertani (LB) (CONDA, Madrid, Spain), in order to revitalize them before use for microbiological assay.

### 3.4. In Vitro Antibacterial Assays

#### 3.4.1. Agar Well Diffusion Method

A preliminary in vitro antimicrobial assay was performed in order to qualitatively evaluate the TEO antibacterial activity against tested foodborne isolates. Specifically, the agar well diffusion method was carried out, similar to Perez (1990) [61]. Briefly, bacterial meat isolates were grown in LB broth. After reaching an optical density (O.D.) of 0.5 OD_600nm_, aliquots (200 µL) of each microbial suspension were spread on LB agar. Then, wells of 6 mm diameter were punched with sterilized glass Pasteur into agar media and were filled up with TEO aliquots (25, 50, 100 µL) and with selected test controls. In particular, gentamicin (Sigma-Aldrich S.r.l., Milano, Italy) and amoxicillin (Sigma-Aldrich S.r.l., Milano, Italy) were used as positive controls for *S.* Typhimurium ST1 and *B. cereus* BC3 isolates, respectively; distilled water was used as a negative control. After plates incubation at 37 °C for 24 h, in aerobic conditions, the mean diameter of the inhibition zone (MDIZ) (expressed in mm) produced by TEO around the wells was measured in order to evaluate the TEO in vitro antibacterial activity against the selected bacterial foodborne pathogens.

#### 3.4.2. Tube Dilution Method

Tube dilution method with standard bacterial inoculum 1 × 10^5^ CFU/mL (Colonies Forming Units/mL) in LB broth was performed, according to the Clinical and Laboratory Standards Institute (CLSI) 2022 guidelines [62], in order to quantitatively determine the susceptibility of *S.* Typhimurium ST1 and *B. cereus* BC3 foodborne isolates to different concentrations of TEO. In brief, TEO was directly added to the broth medium reaching increasing final concentrations (0, 5, 10, 20, 40, 60, 80, 100, 120, 140 μL mL^−1^). After a vigorous stirring by vortex mixer, tubes were incubated at 37 °C for 24 h in aerobic conditions, with constant shaking to maintain homogenous TEO micelle aggregates in the broth medium. After incubation, the values of minimum inhibitory concentration (MIC) were determined through the observation of tube turbidity. In addition, for the determination of the minimum bactericidal concentration (MBC) values, aliquots of serial dilutions of the bacterial suspensions were spread on LB agar and viable bacterial counts were performed after plate incubation under appropriate growth conditions. MIC was assigned to the lowest concentration of TEO that prevented the bacterial growth. MBC was defined as the minimum concentration of TEO that killed 99% of bacteria from the initial inoculum. Gentamicin (Sigma-Aldrich S.r.l., Milano, Italy) and amoxicillin (Sigma-Aldrich S.r.l., Milano, Italy) were used as positive controls for *S.* Typhimurium ST1 and *B. cereus* BC3 isolates, respectively, while distilled water was used as the negative control.

### 3.5. In Vitro Antibiofilm Assays

#### 3.5.1. Tissue Culture Plate Method (TCPM)

The so-called Tissue culture plate method (TCPM) was carried out to measure biofilm biomass of tested foodborne bacteria, as described in Sateriale et al. (2020) [63]. After growing overnight cultures of selected microorganisms at 37 °C for 24 h in LB broth, with aerobic incubation, the OD_600nm_ of fresh cultures were further adjusted until 0.5 OD. Then, aliquots of starter cultures (200 µL) were dispensed into wells of a 96-well flat-bottomed microplate (Nunc™ MicroWell 96-well microplates, Thermo Scientific, Roskilde, Denmark) in six replicates, using sterile LB broth as the negative control. The incubation at 37 °C for 24 h without shaking allowed the adhesion of bacterial cells to the microplate surface. Each bacterial culture was removed after incubation, and free-floating bacterial cells were removed by washing wells with phosphate buffer saline (1× PBS, pH 7.3) three times. Then, adherent biofilms were fixed with 85% ethanol (Sigma-Aldrich, Merck KGaA, Darmstadt, Germany) for 15 min and stained with 0.2% crystal violet (Sigma-Aldrich, Merck KGaA, Darmstadt, Germany) for 5 min. After washing with deionized water to remove the stain excess, plates were dried upside down in a thermostat at the temperature of 30 °C for 10 min. After the addition of 85% ethanol (Sigma-Aldrich, Merck KGaA, Darmstadt, Germany) in crystal violet-stained biofilms, the OD values were recorded at 600 nm wavelength by a microplate reader (Bio-Rad Microplate reader, Model 680). The measurement of the optical density of bacterial isolates (OD_I_), and the comparison with values measured for sterile LB broth used as the negative control (OD_C_), allowed to classify the bacterial isolates as non-adherent (OD_I_ ≤ OD_C_), weakly adherent (OD_C_ < OD_I_ ≤ 2 × OD_C_), moderately adherent (2 × OD_C_ < OD_I_ ≤ 4 × OD_C_) and strongly adherent (4 × OD_C_ < OD_I_).

#### 3.5.2. Biofilm Formation Inhibition Assay

To evaluate the ability of TEO to inhibit biofilm formation, some modifications to the tissue culture plate method were carried out. In particular, increasing concentrations of TEO (0, 10, 20, 40, 80, 100 µg mL^−1^) were added to each 96-well microplate well. Sterile LB broth was added to the negative control wells. Finally, 0.1 mL of each bacterial culture, with 0.5 OD A_600nm_ wavelength, was pipetted to each well, reaching the final volume of 0.2 mL. LB (0.2 mL) broth was added into the wells without bacterial culture as the blank. The plates were wrapped loosely and aerobically incubated at 37 °C for 24 h without shaking. After incubation, the staining step of the TCPM method with crystal violet was performed, followed by microplate reading at the wavelength of 600 nm. According to Bakkiyaraj et al. (2013) [64], results were indicated as the percentage of biofilm formation inhibition, as follows:

$$\mathrm{Biofilm}\;\mathrm{formation}\;\mathrm{inhibition}\;\%=\left(\frac{OD_{control}-OD_{assay}}{OD_{control}}\right)\;\times 100$$

where $OD_{control}$ corresponds to the mean optical density measured for bacterial biofilms grown in the absence of TEO, while $OD_{assay}$ is the mean optical density measured for bacterial biofilms grown in the presence of TEO. Minimum Biofilm Inhibition Concentration (MBIC) was defined as the lowest concentration of TEO able to produce bacterial biofilm inhibition.

### 3.6. Statistical Data Analysis

Antimicrobial assays were performed against three independent bacterial cultures. Obtained results have been graphically reported by ‘GraphPad Prism 7.00′ software and statistical significance has been validated by one-way ANOVA test, with Dunnett’s and Tukey’s corrections. *p* values < 0.05 have been considered statistically significant.

## 4. Conclusions

In this study, we demonstrated that thyme essential oil (TEO) has an excellent antibacterial activity against important food pathogens, *Salmonella enterica* subsp. *enterica* serovar Typhimurium and *Bacillus cereus*, in planktonic form. The bacteriostatic and bactericidal activities of TEO were higher against the Gram-positive pathogens than the Gram-negative ones. TEO was also active in counteracting the biofilm formation by *S.* Typhimurium and *B. cereus*. Altogether, the results of this study suggest that TEO may be a viable candidate as a natural food additive to be employed in the food industry, as an alternative to synthetic preservatives, to control foodborne contamination and biofilm formation associated with *S.* Typhimurium and *B. cereus* pathogens. The antibacterial and antibiofilm activities of TEO against meat pathogen isolates stimulate further research to assess TEO ability to prolong the shelf-life of meat food.

## Figures and Tables

**Table 1:** Chemical composition of thyme essential oil.

N° Peak	RI	Relative Peak Area %	Identified Compound	Structure
1	973	1.232 ± 0.01	sabinene	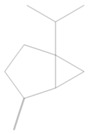
2	978	7.177 ± 0.04	β-pinene	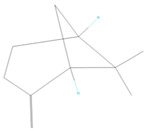
3	996	0.518 ± 0.00	α-phellandrene	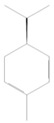
4	1021	1.680 ± 0.50	o-cymene	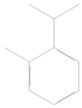
5	1035	39.391 ± 0.20	limonene	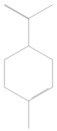
6	1064	4.405 ± 0.02	γ-terpinene	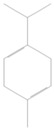
7	1302	44.435 ± 0.22	thymol	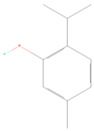

Results are reported as mean values of relative peak area % (area peak compound/area peak total compounds)×100) ± standard deviation (n = 2). Constituents are presented in the order of elution from the HP-5MS column. NIST MS Search Software (version 2.0) and Wiley 8 mass spectral libraries (USA National Institute of Science and Technology software 2.0) of the GC/MS data system allowed the identification of compounds. The 2D structures were acquired from PubChem Compound Database. RI, retention index.

**Table 2:** Values of minimal inhibitory concentration and minimal bactericidal concentration of thyme essential oil and positive controls against *S.* Typhimurium ST1 and *B. cereus* BC3 food isolates.

Antibacterial Agent	*S.* Typhimurium ST1		*B. cereus* BC3	
	MIC	MBC	MIC	MBC
**TEO**	20 µL mL^−1^	100 µL mL^−1^	10 µL mL^−1^	80 µL mL^−1^
**GNT**	30 μg mL^−1^	500 μg mL^−1^	-	-
**AMX**	-	-	50 μg mL^−1^	200 μg mL^−1^

MIC, minimum inhibitory concentration; MBC, minimum bactericidal concentration; TEO, thyme essential oil; GNT, gentamicin; AMX, amoxicillin.

## Data Availability

Not applicable.

## Supplementary Materials

The following supporting information can be downloaded at: https://www.mdpi.com/article/10.3390/antibiotics12030485/s1, Table S1. Antimicrobial susceptibility profile of *S.* Typhimurium ST1 and *B. cereus* BC3 food isolates.
