# Supplementary material for: Antibacterial and Antibiofilm Efficacy of Thyme (Thymus vulgaris L.) Essential Oil against Foodborne Illness Pathogens, Salmonella enterica subsp. enterica Serovar Typhimurium and Bacillus cereus
Source: Antibiotics (Basel). 2023 Feb 28;12(3):485. doi: 10.3390/antibiotics12030485 (PMC10044538; doi:10.3390/antibiotics12030485)
Supplement: Supplementary file 1 [file antibiotics-12-00485-s001.zip › antibiotics-2139082-supplementary.pdf]

Table S1. Antimicrobial susceptibility profile of *S.* Typhimurium ST1 and *B. cereus* BC3 food isolates.

**Antibacterial agents (1 mg mL^{-1})**	**MDIZ (mm)**	
	***S.* Typhimurium ST1**	***B. cereus* BC3**
**AMX**	12.01 ± 0.01 [a]	23.93 ± 0.74 [a]
**AMP**	10.67 ± 0.47 [a]	15.50 ± 0.41 [b]
**CAF**	12.67 ± 0.47 [a]	11.33 ± 0.47 [c]
**GNT**	16.38 ± 0.85 [b]	8.50 ± 0.41 [d]

[1] MDIZ, mean diameter of the inhibition zone; AMX, amoxicillin; AMP, ampicillin; CAF, chloramphenicol; GNT, gentamicin.

[2] Results were obtained by agar well diffusion method; triplicate assays with independent cultures. The mean diameters of inhibition zone (in mm) is reported as mean values ± standard deviation.

[3] One-way ANOVA test with Tukey's post hoc test ($p<0.05$) was performed to evaluate statistical significance for multiple comparisons for each bacterial isolate. Different letters (a, b, c, d) indicate significant differences.
